# BET inhibition unmasks a targetable glycolytic dependency through a HIF1α stabilization and driven transcriptional program in a defined subset of triple-negative breast Cancer

**DOI:** 10.1038/s41420-026-03230-8

**Published:** 2026-07-08

**Authors:** Teresa Rossi, Egidio Iorio, Mattea Chirico, Maria Elena Pisanu, Federica Torricelli, Alessia Ciarrocchi, Mariaelena Pistoni

**Affiliations:** 1 Laboratory of Translational Research, Azienda USL-IRCCS di Reggio Emilia, Reggio Emilia, Italy; 2 High Resolution NMR Unit, Core Facilities, Istituto Superiore di Sanità, Rome, Italy

**Keywords:** Transcription, Cancer metabolism

## Abstract

Triple-negative breast cancer (TNBC) is characterized by transcriptional and metabolic heterogeneity, which influences its response to therapeutics. Epigenetic drugs such as Bromodomain and Extra-Terminal domain inhibitors (BETi) are no exception to this variable response. However, the determinants of BETi sensitivity and the underlying mechanisms of response remain poorly understood, particularly in the context of metabolic reprogramming. Here, we investigated the responses to the BETi JQ1 and OTX015 across a heterogeneous panel of TNBC models. We found that the susceptibility to BETi partially correlates with basal BRD4 protein levels, but only in contexts characterized by high baseline cMYC levels, where BETi treatment triggers an upregulation of glycolytic genes, an effect that is absent in models displaying an intrinsically glycolytic phenotype. While the glycolysis inhibitor 2-deoxy-D-glucose (2-DG) is effective as a single agent in the glycolytic-prone setting, it has limited efficacy in other TNBC models. Strikingly, a notable additive effect is visible when BETi are combined with 2-DG, leading to significant apoptotic induction, specifically in the BETi-responsive cells, whereas this additive effect is not observed in the glycolysis-driven models. Mechanistically, we identified that BETi induces the HIF1α transcriptional program in cMYC-high cells, which upregulates key glycolytic enzymes. HIF1α depletion reduced this response, confirming that HIF1α is functionally required for this adaptive rewiring. In conclusion, TNBC cells adapt to BETi by undergoing HIF1α-mediated metabolic rewiring towards glycolysis. This adaptive response creates a vulnerability, rendering these tumors sensitive to the combination of BET and glycolysis inhibitors. By mapping a transcriptional-metabolic axis that dictates BETi sensitivity, this study moves beyond the identification of a resistant subset of TNBC to reveal a deeper principle: targeted inhibition can actively reprogram cellular circuitry, thereby constructing its own unique therapeutic vulnerability. Thus, the path to overcoming resistance may lie not in evading this rewiring, but in strategically exploiting the alternative dependencies it creates.

## Introduction

Triple-negative breast cancer (TNBC) is an aggressive subtype characterized by the absence of estrogen receptor (ER), progesterone receptor (PR), and HER2 amplification. It accounts for 15–20% of breast cancers and is associated with a high risk of recurrence and metastasis, highlighting the need for novel treatments. Cancer is not only driven by genetic alterations but also by metabolic changes. A key hallmark of cancer, including TNBC, is metabolic reprogramming [1], often involving a shift toward aerobic glycolysis (the Warburg effect) [2, 3], even in the presence of oxygen, to support uncontrolled growth and proliferation and adapt to the tumor microenvironment [4, 5]. Consistent with this model, TNBC exhibits higher glycolytic activity than estrogen receptor-positive (ER+) breast cancer [6–8], underscoring how its metabolic plasticity represents both a therapeutic challenge and a potential vulnerability [9–11]. Bromodomain and extra-terminal domain (BET) proteins, including BRD2, BRD3, and BRD4, function as epigenetic readers that regulate chromatin structure and gene transcription. They bind to acetylated histones, linking epigenetic states to gene expression, and facilitate transcriptional elongation, particularly at enhancer regions. BET inhibitors (BETi) represent a class of epigenetic drugs that have shown preclinical promise in cancers like TNBC by targeting epigenetic readers regulating oncogenic transcription. However, their clinical application as monotherapies has been hampered by dose-limiting toxicities, particularly hematological events such as thrombocytopenia and anemia, as well as gastrointestinal adverse effects [12–15]. Thus, there is a compelling need to explore optimized dosing regimens and rational drug combinations to maximize the therapeutic potential of BET inhibitors. The rationale for prioritizing BET inhibitors in TNBC is its distinct molecular profile. TNBC exhibits elevated expression of BET-dependent genes and a pronounced reliance on specific, targetable oncogenic pathways [14]. Furthermore, TNBC cells display a heightened dependence on BET proteins, particularly BRD4, for survival and proliferation [14, 16–18]. BETi also significantly impact cellular metabolism [19–22], yet how metabolic heterogeneity dictates BET inhibitor response remains unclear. Our prior work demonstrated that BETi modulates lipid metabolism and mitochondrial function in TNBC models [23, 24].

This study investigates how basal transcriptional and metabolic states determine TNBC cell fate following BET inhibition, ultimately considering a rationale for a combination strategy. We test the hypothesis that co-targeting simultaneously epigenetic and metabolic dependencies, specifically by combining a BETi with the glycolysis inhibitor 2-deoxy-D-glucose (2-DG), will enhance efficacy, particularly in BRD4/cMYC-high subtypes. Mechanistically, we identify HIF1α stabilization, a master regulator of tumor adaptation and aggressiveness, as a potential driver of these compensatory adaptations. Together, this study provides insight into BETi-induced metabolic adaptation, aiming to inform the development of metabolically informed combination therapies in the treatment of TNBC.

## Results

### BRD4 and cMYC basal expression levels associate with sensitivity to BETi in breast cancer cells

**Fig. 1:**
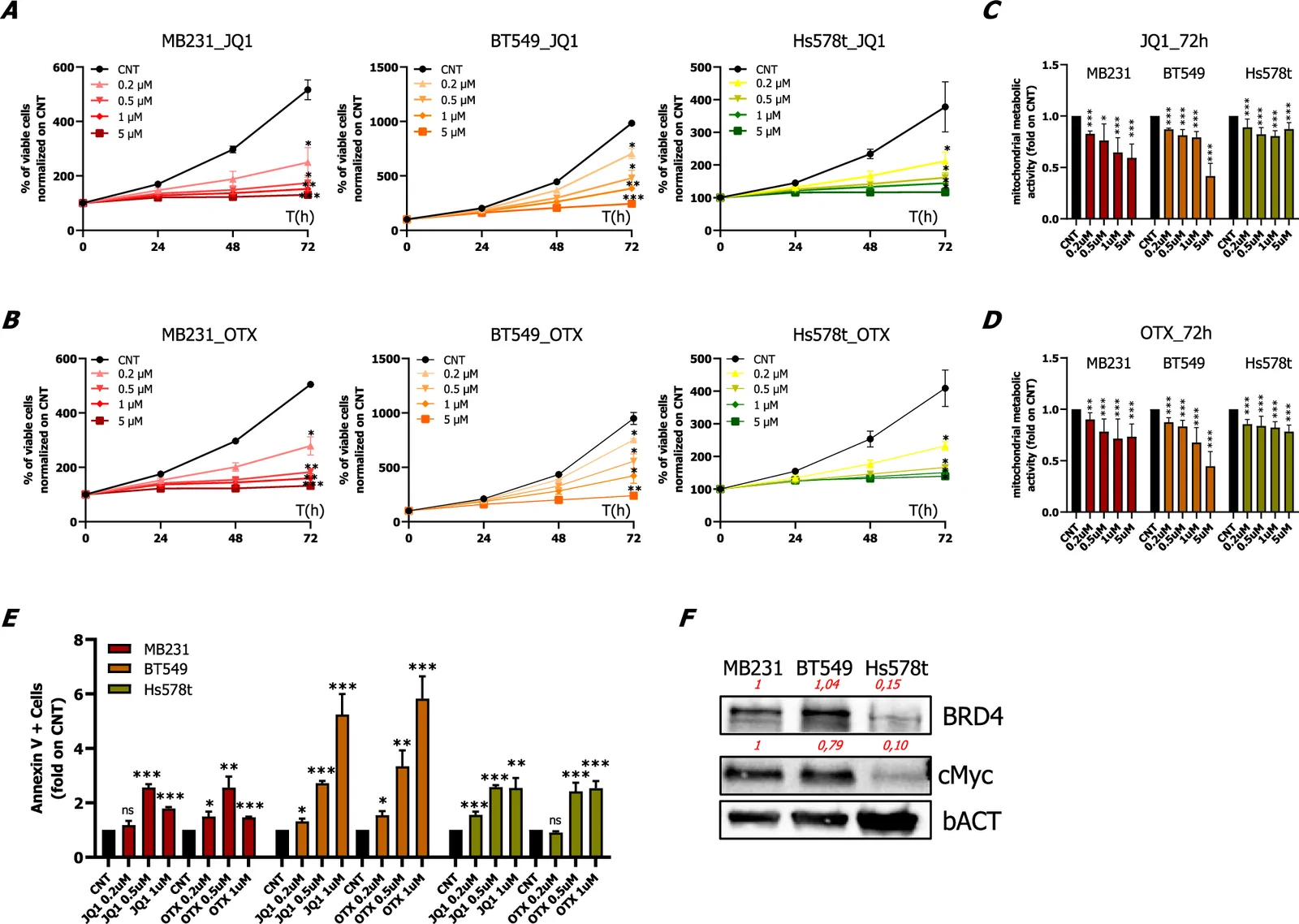
BET inhibitor sensitivity correlates with BRD4 levels in cMYC-high TNBC models. Cell proliferation assay (Incucyte) on TNBC cells using different concentrations (CNT, 0.2 μM, 0.5 μM, 1 μM, and 5 μM) of JQ1 (**A**) or OTX (**B**) over 72 hours of treatment (T(h)). MTT assay on TNBC cells treated with JQ1 (**C**) or OTX (**D**) for 72 hours. The results are presented as fold change of metabolic viability normalized to the control (CNT). BETis were used at a final concentration of 0.2 μM, 0.5 μM, 1 μM, and 5 μM. **E**. Percentage of Annexin V positive (+) cells in TNBC cells treated with 0.2 μM, 0.5 μM and 1 μM of JQ1 or OTX for 72 hours expressed as fold on the respective control (CNT). **F** Western blot images on TNBC cells probed with antibodies against BRD4, cMYC, and β-Actin (bACT). In red, the band quantification for both antigens relative to the respective housekeeping (bACT).

To characterize the cellular responses to BET inhibition beyond simple growth suppression, we assessed the dose- and response-dependent effects using two structurally distinct BET inhibitors: JQ1 and OTX015 (hereafter referred to as OTX), in a panel of breast cancer cell lines. We focused on three claudin-low TNBC models: MDA-MB231 (MB231), BT549, and Hs578t, with intermediate responses to chemotherapy [25–27] and the luminal A MCF7 cell line as a hormone receptor-positive control to account for breast cancer heterogeneity. In these settings, we tested cell proliferation, mitochondrial metabolic activity, and cell death using different doses of BETi (ranging from 0 to 5 µM) for up to 72 hours of treatment. Live-imaging Incucyte analysis revealed that both JQ1 and OTX significantly reduced cellular proliferation in all tested cell lines (Fig. 1A, B and Supplementary Fig. 1A). Among the TNBC models, Hs578t cells exhibited the highest sensitivity to growth inhibition, followed by MB231, while BT549 cells showed a comparatively attenuated proliferative response (Fig. 1A, B). MCF7 cells displayed growth inhibition profiles comparable to those observed in TNBC cell lines (Supplementary Fig. 1A). We next evaluated mitochondrial metabolic activity as a readout integrating cellular metabolic function and membrane integrity. BETi treatment induced a significant dose-dependent reduction in mitochondrial activity in MB231 and BT549 cells, whereas Hs578t cells exhibited a more modest, though statistically significant, decrease (Fig. 1C, D). In contrast, MCF7 cells were highly sensitive to BETi in this assay (Supplementary Fig. 1B). Consistent with these observations, BETi treatment induced apoptotic cell death across the TNBC panel, although with distinct patterns. BT549 cells showed a progressive, dose-dependent induction of apoptosis compared to untreated controls (CNT) (Fig. 1E). Altogether, while Hs578t cells exhibited the most pronounced reduction in total cell number (Fig. 1A), MB231 and BT549 displayed a more robust induction of apoptosis and mitochondrial dysfunction (Fig. 1C–E), suggesting that the sensitivity to BET inhibition is not uniform across phenotypic outputs. To investigate whether these heterogeneous responses were associated with basal BET pathway activity, we quantified BRD4 and cMYC protein levels. Both BRD4 and cMYC expression varied substantially among the TNBC cell lines (Fig. 1F). Notably, MB231 and BT549 cells exhibited higher basal cMYC expression compared to Hs578t cells, suggesting that BRD4–cMYC axis activity may influence the qualitative nature of the response to BET inhibition. Treatment with BETi downregulates cMYC expression (Supplementary Fig. 1C) in all the TNBC cell lines, demonstrating target engagement under the experimental conditions. However, given the high basal expression of BRD4 and cMYC in MB231 and BT549 (Fig. 1F), we focused on these lines to investigate the metabolic adaptations to BETi. Importantly, these data indicate that basal BRD4 and cMYC levels do not strictly predict overall sensitivity to BET inhibition, but rather shape specific phenotypic outcomes.

### BETi induces a delayed upregulation of glycolytic genes in cMYC-high TNBC cells

**Fig. 2:**
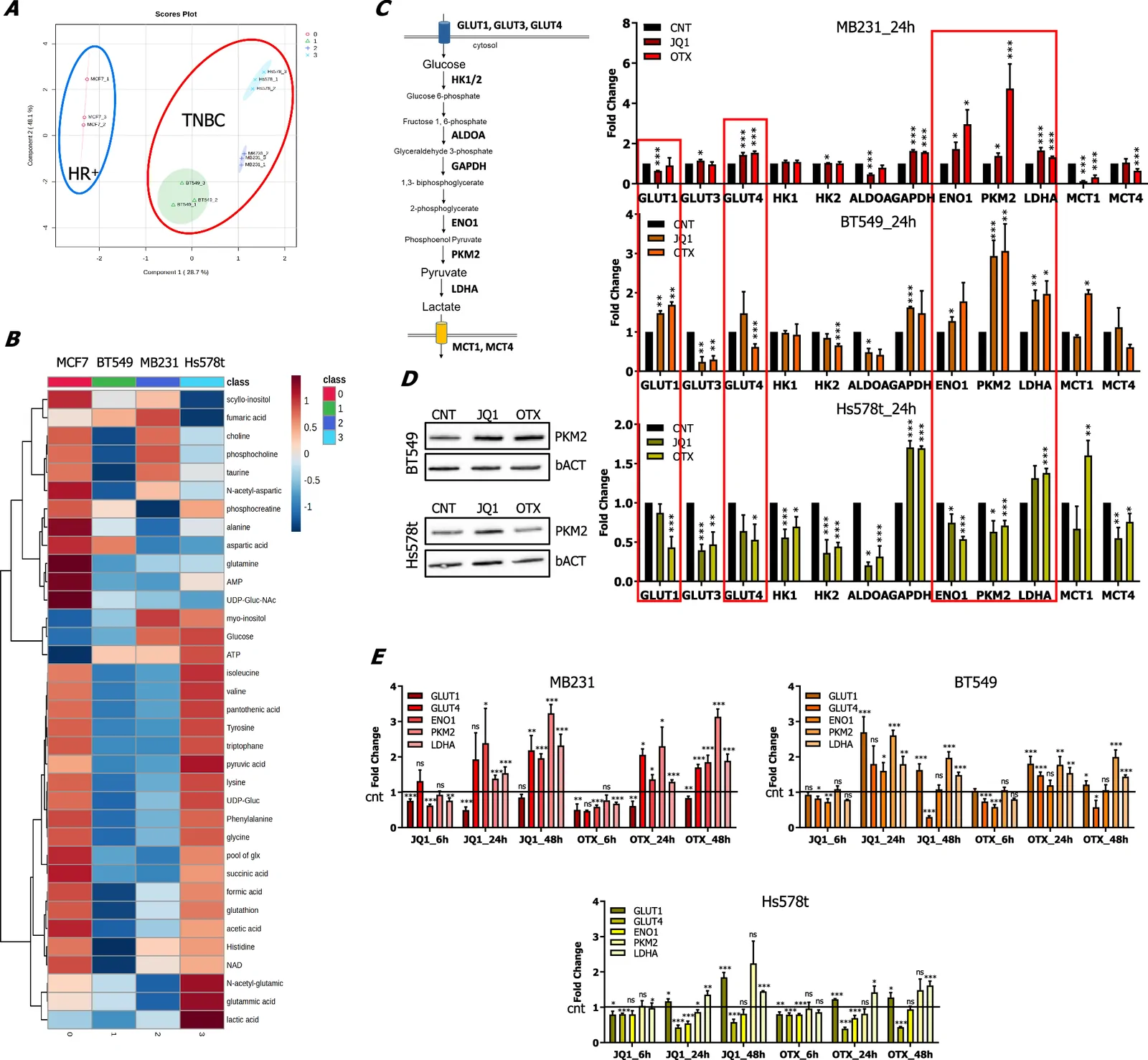
BETi induces a glycolytic transcriptional program in non–glycolysis-intrinsic TNBC cells. Partial Least Squares-Discriminant analysis (PLS-DA; Q^2^ = 0.6) score scatter plots (**A**) and heatmap (**B**) obtained for the three TNBC cells and hormone receptor-positive cancer cells (0 = MCF7; 1 = BT549; 2 = MB231; 3 = Hs578t (*n* = 3)). In the heatmap, columns illustrate each sample group and rows correspond to the mean normalized peak area of each metabolite, ranging from the minimum value (dark blue) to the maximum value (dark red). **C** Left, a schematic representation of the glycolytic pathway. Key glycolytic enzymes (HK1/2, ALDOA, GAPDH, ENO1, PKM2, and LDHA) are shown, with glucose ultimately converted to lactate. Right, qRT-PCR analysis of glycolytic enzyme expression in MB231, BT549, and HS578t after 24 hours of BETi administration. Data are expressed in fold change versus the control (CNT). The red boxes are the genes analyzed in (**E**). **D** Western blot images of BT549 (top) and Hs578t (bottom) cells probed with antibodies against PKM2 and β-Actin (bACT). **E** qRT-PCR analysis of a selection of glycolytic enzymes in MB231, BT549, and HS578t after 6, 24, and 48 hours of BETi administration. Data are expressed in fold change versus control, represented as the black line.

Given our previous observations that BETi influences metabolic pathways and metabolite levels in TNBC cells [23, 24], we investigated whether basal metabolic states influence cellular responses to BET inhibition. We first characterized the intrinsic metabolic profiles of MB231, BT549, Hs578t, and MCF7 cells using Nuclear Magnetic Resonance (NMR) spectroscopy. This approach provided a comprehensive overview of the metabolites present in these cell lines under basal conditions. Unsupervised Principal Component Analysis (PCA) and supervised Partial Least Squares Discriminant Analysis (PLS-DA) revealed clear stratification of the breast cancer (BC) cell lines based on their distinct aqueous metabolite profiles (Fig. 2A). The PLS-DA model exhibited strong predictive performance (Q² > 0.6; Supplementary Fig. 1D), and heatmap visualization confirmed distinct metabolic signatures (Fig. 2B). As shown in Fig. 2B, MB231 and BT549 share similarities in the majority of polar metabolites, whereas MCF7 displays a markedly different profile, especially in metabolites such as alanine (ala) and adenosine triphosphate (ATP). Furthermore, lactate (lac) exhibits a marked presence in HS578t compared to the other cell lines. To note, glutamate, lac, and ATP showed significant differences in abundance across the cell lines, suggesting underlying variations in energy metabolism and biosynthetic pathways that may influence their response to BETi. Additionally, metabolites associated with glycolysis (e.g., lac) and the tricarboxylic acid cycle (TCA) (e.g., fumarate, succinate, aspartic acid) tended to cluster together, indicating coordinated regulation (Fig. 2B). PLS-DA loading plots helped identify the most discriminative metabolites, with variable importance in projection (VIP) scores > 1. We selected ten metabolites with VIP > 1 (Supplementary Fig. 1E), highlighting statistically significant changes, and visualized them through a heatmap showing their relative concentrations across the four cell lines. Intracellular metabolomic analysis revealed that Hs578t cells exhibit the highest levels of glucose, pyruvic acid, and lactic acid among the four BC cell lines analyzed, indicative of a glycolytic phenotype. To further investigate glycolytic activity, we quantified the lactate-to-alanine (Lac/Ala) ratio in the extracellular medium of cells cultured over 3, 12, 24, 48, and 72 hours, as a proxy for glycolytic flux. At 72 h, MB231 and BT549 exhibit similar ratios (1.95 and 1.88, respectively), while Hs578t cells show higher Lac/Ala values (3.16) at the same time point, consistent with prior findings [8], suggesting a stronger reliance on glycolysis (Supplementary Fig. 1F). Overall, these findings underscore significant metabolic heterogeneity among BC cells, which prompted us to evaluate whether BET inhibition would affect the expression of glycolytic genes (Fig. 2C). We next ascertain if BET inhibition alters the expression of glycolytic genes. Unexpectedly, treatment with JQ1 or OTX induced a pronounced upregulation of key glycolytic genes, including GLUT1, GLUT4, ENO1, PKM2, and LDHA, in MB231 and BT549 cells (red box in Fig. 2C). In contrast, Hs578t cells showed either no induction or a reduction of these genes, while MCF7 cells exhibited an intermediate response (Fig. 2C and Supplementary Fig. 2A). Western blot analysis confirmed PKM2 upregulation in BT549 cells, whereas PKM2 levels remained unchanged in Hs578t cells, indicating that a residual glycolytic activity is maintained (Fig. 2D and quantification in Supplementary Fig. 2B). The PKM2 protein levels are unchanged in MB231 (data not shown). Time-course analysis showed that glycolytic gene induction in MB231 and BT549 cells was not immediate but became detectable after 24 hours of BETi treatment, with no similar changes in Hs578t cells (Fig. 2E), indicating a delayed adaptive response rather than an acute transcriptional effect. Collectively, these data demonstrate that BET inhibition induces a compensatory glycolytic transcriptional program selectively in cMYC-high TNBC cells lacking an intrinsically glycolytic phenotype.

### Basal metabolic phenotype determines sensitivity to the glycolysis inhibitor 2-deoxy-D-glucose

**Fig. 3:**
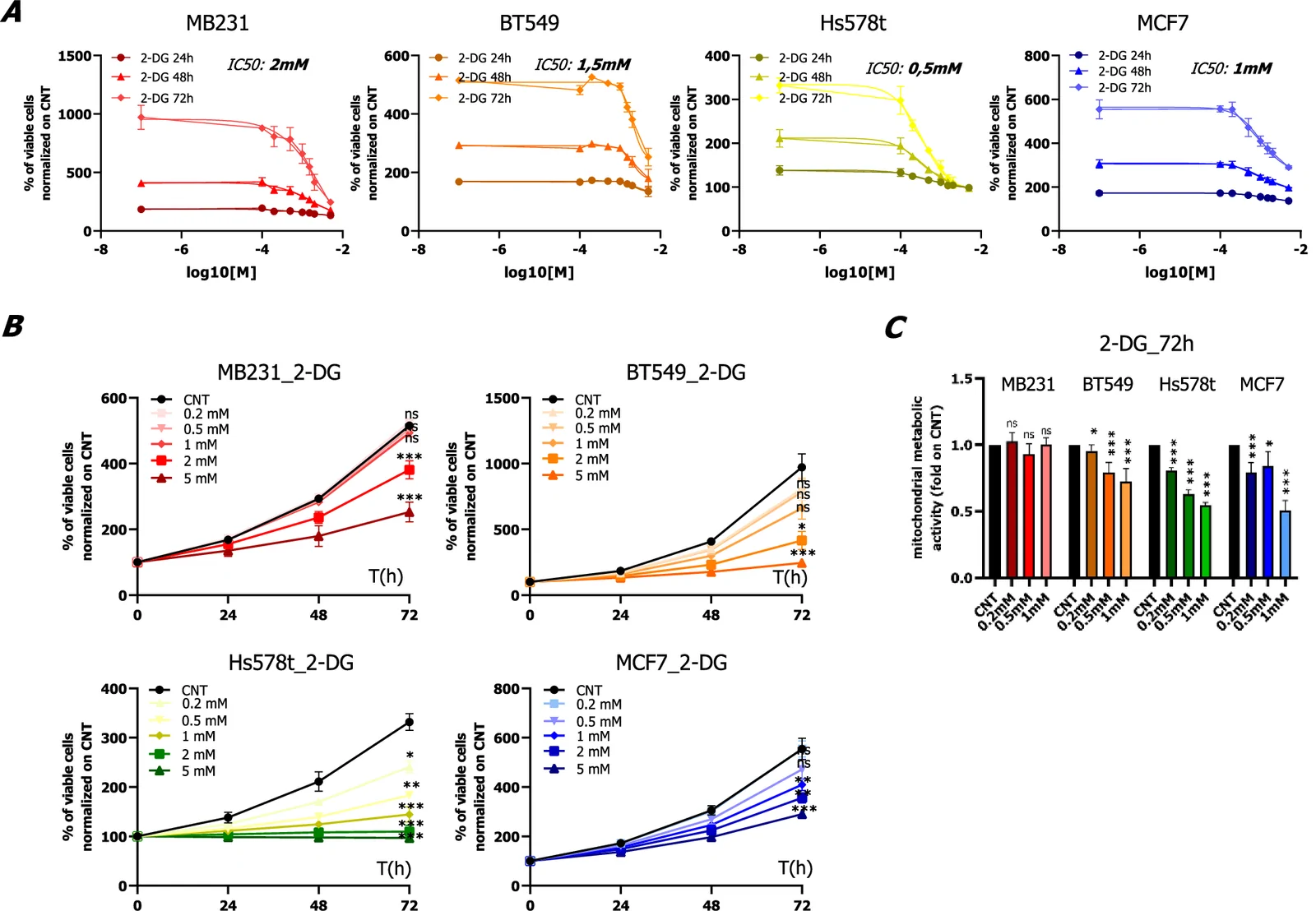
Basal glycolytic dependency determines sensitivity to 2-DG in breast cancer cells. **A** IC50 values of 2-DG in BC cells for 24, 48, and 72 hours, determined via Incucyte. All data were expressed as log10 [M]. The calculated IC50 for each cell line is indicated in the graph. **B** Cell proliferation assay (Incucyte) on BC cells using different concentrations (0 - CNT - 0.2, 0.5, 1, 2 and 5 mM) of 2-DG over 72 hours of treatment. **C** MTT assay on breast cancer cells treated with CNT - 0.2 mM, 0.5 mM, and 1 mM of 2-DG for 72 hours. The results are presented as a fold change of metabolic viability normalized to the relative control (CNT).

Based on the metabolic heterogeneity and pathway dependencies identified above, we next assessed the IC50 of the glycolysis inhibitor 2-DG on BC cells [28, 29], using the same range of concentrations and the same timing. As anticipated, the BC cell lines exhibited a distinct range of sensitivities to 2-DG (Fig. 3A). Hs578t cells, characterized by a strong basal glycolytic bias [8], were the most sensitive to 2-DG (Fig. 3B, C). Conversely, MB231 and BT549 cells, which display less reliance on glycolysis, displayed reduced susceptibility to 2-DG, while MCF7 cells showed an intermediate response consistent with their metabolic profile. These findings indicate that intrinsic metabolic profiling could reveal metabolic vulnerabilities.

### BETi and 2-DG combination induces additive anti‑tumoral effects, predominantly through apoptosis, in a subset of TNBC cells

**Fig. 4:**
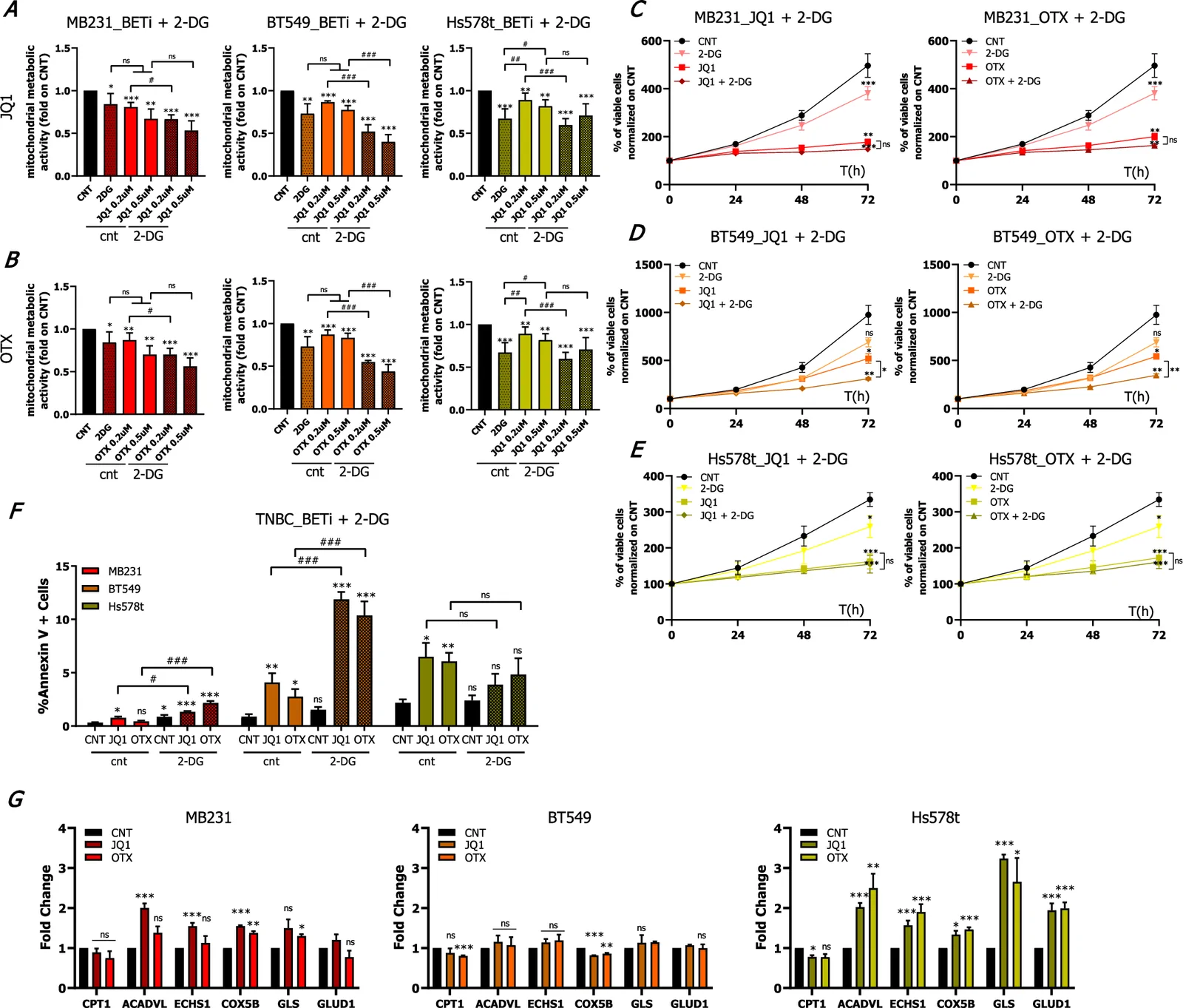
Combined BET and glycolysis inhibition selectively induces apoptosis in BETi-sensitive TNBC cells. MTT assay on TNBC cells treated with BETi (JQ1 (**A**) or OTX (**B**)) and 2-DG, alone or combined, for 3 days. The results are presented as a fold change of metabolic activity normalized to the control (CNT). BETi were used at a final concentration of 0.2-0.5 μM and 2-DG at the IC50 relative concentration. Cell proliferation assay (live cell imaging through Incucyte quantification) on MB231 (**C**), BT549 (**D**), and HS578t (**E**) cells to observe the effect of BETi (JQ1 or OTX 0.5 µM) and 2-DG over 72 hours of treatment. BETi and 2-DG were used alone or in combination according to the concentration previously indicated. **F** Percentage of Annexin V positive (+) cells of TNBC cells treated with BETi (0.5 μM) and 2-DG (at the IC50 values) for 3 days. In (**A**, **B**, and **F)**, the significance is calculated versus the CNT-treated cells (indicated with *) or between the treatments (indicated with the #). **G** qRT-PCR analysis of Carnitine Palmitoyltransferase 1A (CPT1a), Acyl-Coenzyme A Dehydrogenase Very Long Chain (ACADVL), Enoyl-CoA Hydratase Short Chain 1 (ECHS1), Cytochrome C Oxidase Subunit 5B (COX5B), GLS and GLUD1 expression in MB231, BT549, and HS578t after 48 hours of BETi administration (0.5 μM). Data are expressed in fold change versus the control (CNT).

**Fig. 5:**
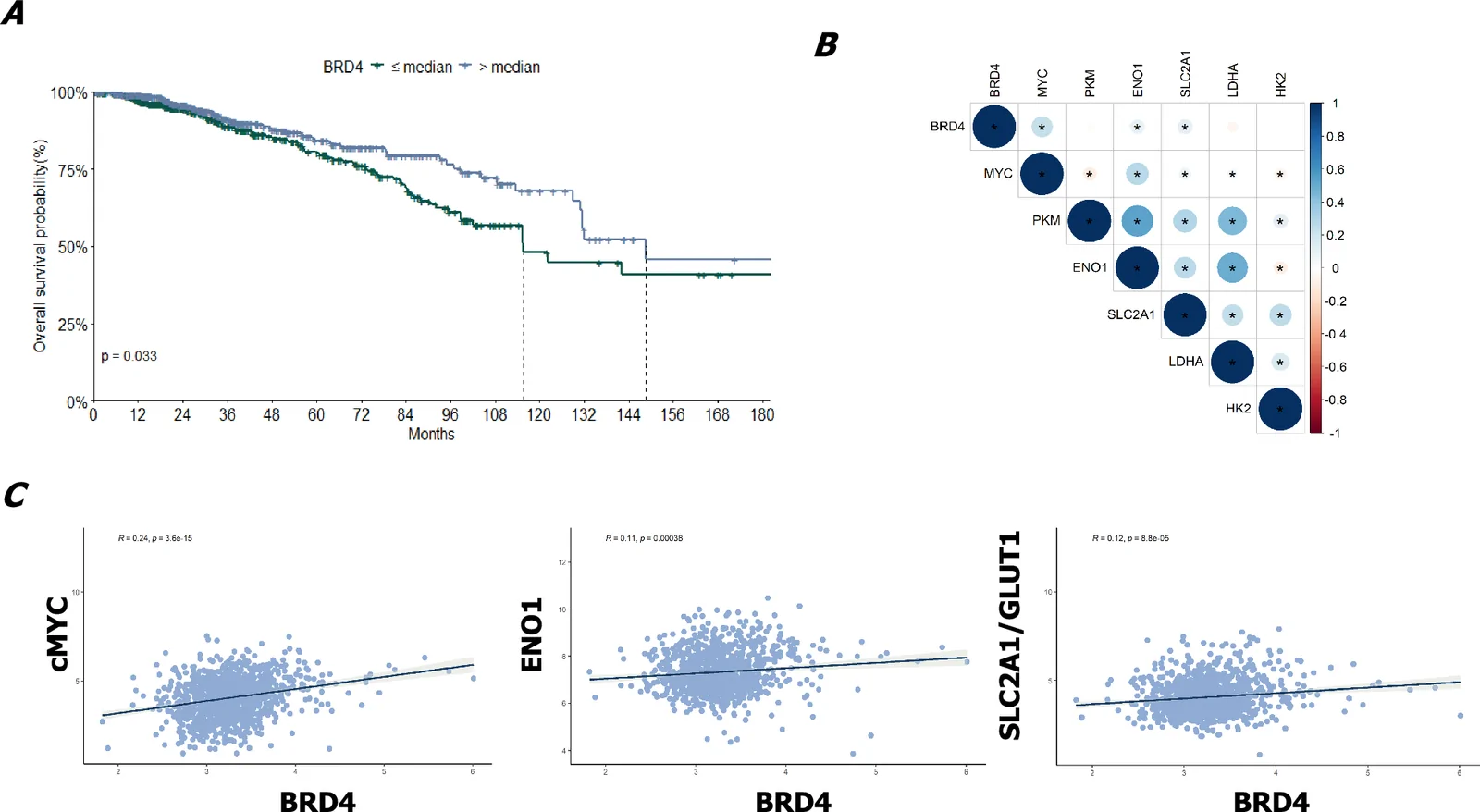
A BRD4–cMYC axis links BET inhibition to adaptive glycolytic rewiring. **A** Kaplan-Meier survival curves generated from the TCGA breast cancer cohort. Patients were stratified into BRD4-high (>median expression) and BRD4-low (≤median expression) groups. A log-rank test was used to determine statistical significance (*p* < 0.05). **B** Correlation plot displaying the pairwise Spearman correlation coefficients between the mRNA expression levels of BRD4, cMYC, and key glycolytic genes (SLC2A1/GLUT1, HK2, ENO1, PKM, LDHA) across the TCGA breast cancer samples. The color intensity and size of the circles correspond to the strength and direction of the correlation, as indicated by the scale bar; asterisks indicate significant correlations (*p* < 0.05). **C** Spearman test correlation of BRD4 and significantly correlated genes: cMyc, ENO1, and SLC2A1/GLUT1.

The metabolic and transcriptional heterogeneity observed among TNBC cells provided a rationale for exploring a combinatorial therapeutic strategy targeting both epigenetic regulation and glucose metabolism. We therefore evaluated the effects of combining BETi (JQ1 and OTX) with the glycolysis inhibitor 2-DG, with the aim of enhancing anti-tumoral efficacy. The combination strategy allows to reduce drugs doses, with clinical benefit for BETi administration, whose clinical application has been limited by systemic toxicity. We first assessed mitochondrial metabolic activity following single-agent and combination treatments. In BT549 cells, the combination of BETi with 2-DG resulted in a significant additive reduction in mitochondrial activity compared to either agent alone (Fig. 4A, B). In MB231 cells, a similar trend was observed, although the effect did not reach statistical significance. In contrast, no cooperative effect was detected in Hs578t cells, where mitochondrial impairment appeared to be largely driven by 2-DG, while BETi contributed minimally (Fig. 4A, B). MCF7 cells displayed a distinct pattern, with proliferation predominantly affected by BETi and mitochondrial activity comparably reduced by both treatments (Supplementary Fig. 2C). Analysis of cell proliferation mirrored these findings. BT549 cells exhibited an additive reduction in proliferation upon combined BETi and 2-DG treatment (Fig. 4D), whereas MB231, Hs578t, and MCF7 cells did not show enhanced growth inhibition beyond that achieved with single agents (Fig. 4C, E and Supplementary Fig. 2D, respectively). Since Hs578t cells are already highly sensitive to both BET inhibition and 2-DG as single agents, we drastically reduced the doses of both drugs; however, without any additive effects (data not shown). These data indicate that Hs578t cells are intrinsically highly sensitive to both BET inhibition and glycolytic interference, likely reflecting a strong dependency on BET-regulated transcriptional programs and glucose metabolism. Strikingly, evaluation of apoptotic cell death revealed a clear and selective additive effect of the BETi + 2-DG combination in MB231 and BT549 cells. Annexin V staining demonstrated a significant increase in apoptosis following combination treatment compared to either agent alone in these two cell lines, whereas no such effect was observed in Hs578t cells (Fig. 4F). In BT549 cells, the reduction in mitochondrial activity strongly correlated with apoptotic induction (r = −0.94, data not shown), indicating that the additive effect of the drug combination is primarily driven by enhanced cell death rather than cytostatic effects. To mechanistically contextualize this selective vulnerability, we integrated these findings with transcriptional and metabolic data. MB231 and BT549 cells express 8–10-fold higher basal levels of cMYC compared to Hs578t cells (Fig. 1F), and BETi treatment induces the upregulation of glycolytic genes specifically in these cMYC-high models (Fig. 2C, D). Moreover, prior Seahorse analysis demonstrated that BETi reduces glycolytic flux by approximately 20% in MB231 cells, compared to nearly 50% in Hs578t cells [23], indicating that glycolysis is only partially suppressed in the BETi-sensitive, cMYC-high context. In contrast, Hs578t cells, which exhibit an intrinsically glycolytic phenotype, fail to benefit from the BETi + 2-DG combination. Instead, these cells appear to engage alternative metabolic survival pathways, as evidenced by the significant upregulation of glutaminolytic enzymes GLS and GLUD1 (Fig. 4G), together with early downregulation of glucose transporters GLUT1 and GLUT4 (Fig. 2C). This suggests a metabolic rewiring toward non-glycolytic dependencies that limits the efficacy of combined glycolysis and BET inhibition. To evaluate the potential clinical relevance of these findings, we analyzed transcriptomic data from the Breast Invasive Carcinoma dataset of The Cancer Genome Atlas (TCGA-BRCA). Although this cohort includes heterogeneous breast cancer subtypes and treatment contexts, tumors with higher BRD4 expression showed an association with improved overall survival (Fig. 5A). In addition, BRD4 expression positively correlated with cMYC and with several glycolytic genes, including ENO1 and the glucose transporter SLC2A1/GLUT1 (Fig. 5B, C). While these observations should be interpreted cautiously, given the mixed composition of the TCGA cohort, they are consistent with the transcriptional relationships observed in our cellular models. Together with our in vitro data, this analysis suggests that a subset of breast tumors may display a BRD4–cMYC transcriptional program associated with glycolytic regulation.

### HIF1α is required for BET inhibitor–induced glycolytic transcriptional reprogramming in cMYC high TNBC cells

**Fig. 6:**
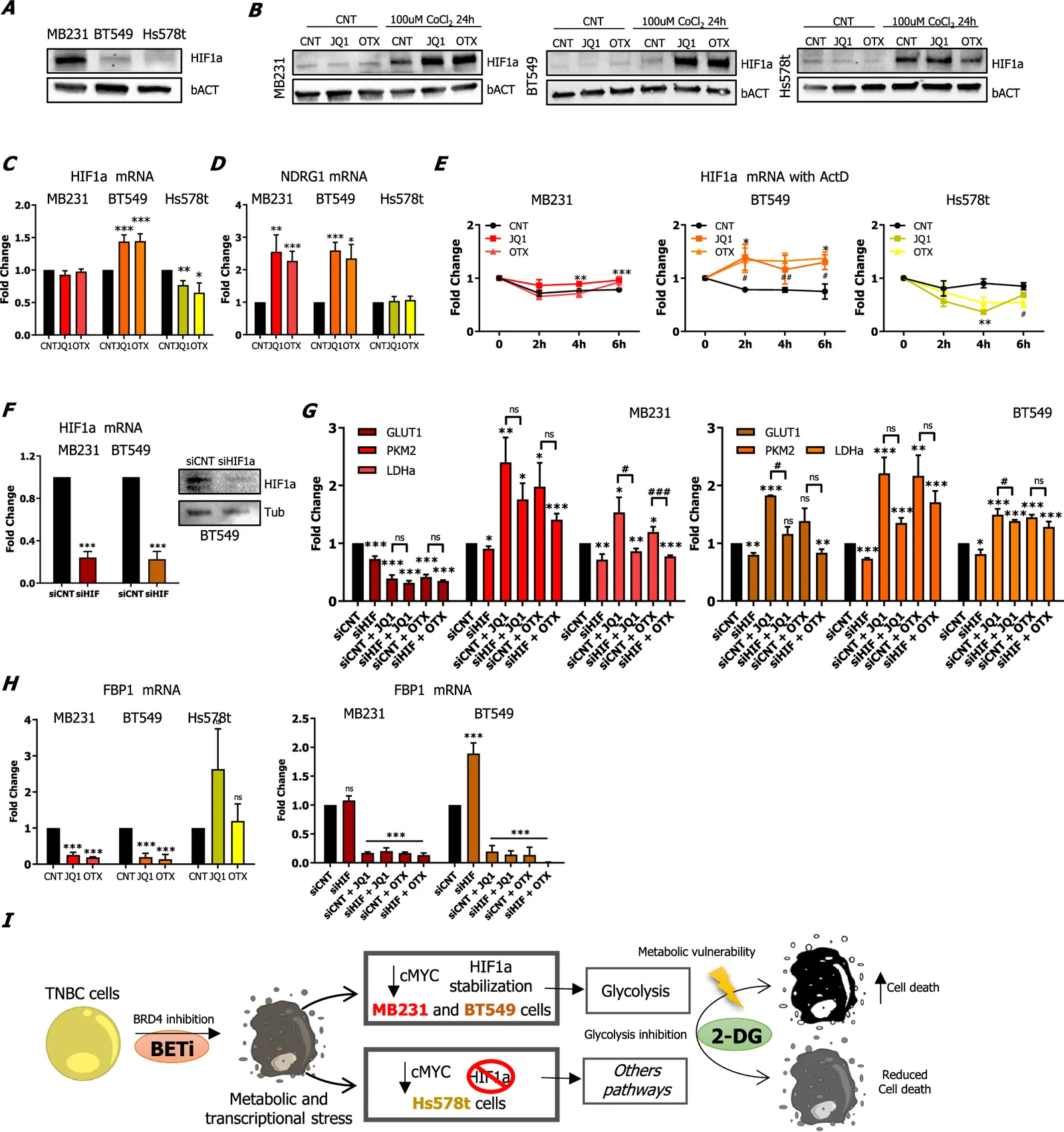
HIF1α is required for BETi–induced glycolytic rewiring in a subset of TNBC cells. **A** Western blot images on TNBC cells probed with antibodies against HIF1a and β-Actin (bACT). **B** Western blot images on TNBC cells treated with 100 μM CoCl_2_ in combination with control (CNT) or BETi (0.5 μM) for 24 hours (24 h) probed with antibodies against HIF1α, and β-Actin (bACT). qRT-PCR analysis of HIF1α (**C**) and NDRG1 (**D**) in MB231, BT549, and HS578t after 24 hours of BETi administration. The significance is calculated versus the CNT-treated cells (indicated with *). **E** qRT-PCR analysis of the HIF1α mRNA in TNBC cells after 24 hours of BETi administration, followed by the indicated time points of actinomycin D (ActD) treatments. The significance is calculated versus the CNT-treated cells (indicated with * for JQ1 treatment and # for OTX treatment) **F** Left, qRT-PCR analysis of HIF1α in MB231 and BT549 cells after 24 hours of siRNA transfection. Right, Western blot images of BT549 cells probed with antibodies against HIF1a and Tubulin (Tub). **G** qRT-PCR analysis of GLUT1, PKM2, and LDHa in MB231 and BT549 cells after 24 hours of siCNT and siHIF1α transfection, treated for 24 hours with control and BETi (JQ1 and OTX). **H** Left, qRT-PCR analysis of FBP1 in MB231, BT549, and HS578t after 24 hours of BETi administration. Right, qRT-PCR analysis of FBP1 in MB231 and BT549 cells after 24 hours of siCNT and siHIF1α transfection, treated for 24 hours with control and BETi (JQ1 and OTX). **I** Graphical abstract.

Although BETi treatment downregulates cMYC across all TNBC cell lines, glycolytic genes (GLUT1, HK2, PKM2, LDHA) are upregulated in MB231 and BT549 cells, but not in Hs578t (Fig. 2C, red boxes). This discrepancy suggests that alternative transcriptional regulators are compensating for cMYC loss. Notably, these glycolytic enzymes are established HIF1α targets, prompting us to investigate HIF1α‘s role in this response. In TNBC cells, HIF1α exhibits variable basal expression (Fig. 6A). Because HIF1α is rapidly degraded under normoxia and often remains below the detection limit of western blot, cells were exposed to CoCl₂ to induce a pseudohypoxic state that stabilizes basal HIF1α levels and enables reliable detection of changes in HIF1α protein abundance. In this pseudohypoxic microenvironment, HIF1α is stabilized and more easily detectable by Western blot in all TNBC cells (Supplementary Fig. 2E). To evaluate whether the levels of HIF1α protein vary among the cell lines upon BETi treatment, we combined JQ1 and OTX with CoCl₂. Interestingly, HIF1α levels increased in MB231 and BT549 cells but failed to upregulate in Hs578t upon BETi addition (Fig. 6B). A similar pattern was observed under basal normoxic conditions (Supplementary Fig. 2F, with relative quantification, on the right). Transcript analysis corroborated this finding: HIF1α mRNA levels were stable in MB231, significantly increased in BT549, and decreased in Hs578t following BETi administration (Fig. 6C). The pseudohypoxic state induced by BETi was further validated by the upregulation of NDRG1, a canonical HIF1α target (Fig. 6D), consistent with our prior RNA-seq data in JQ1-treated MB231 cells (data not shown). To determine whether HIF1α regulation is transcriptional or post-transcriptional, we blocked de novo RNA synthesis with actinomycin D (ActD). While HIF1α mRNA declined in Hs578t, it remained stable in MB231 and high in BT549 (Fig. 6E), suggesting enhanced stability or reduced turnover of HIF1α upon BETi administration and only in the context of cMYC-high cells. As expected, cMYC mRNA (control) sharply decreased after ActD administration (Supplementary Fig. 2G). To establish a role for HIF1α in the BETi–induced glycolytic response, HIF1α was depleted by RNA interference in MB231 and BT549 cells. The efficient target downregulation of HIF1α was confirmed by mRNA levels (Fig. 6F, left), and at the protein level in BT549 cells (Fig. 6F, right). Notably, HIF1α knockdown (KD) reduced basal expression of glycolytic genes, including GLUT1, PKM2, and LDHA, in both MB231 and BT549 cells, indicating that HIF1α contributes to the maintenance of glycolytic gene expression in these models. Importantly, upon BET inhibition, the induction of these glycolytic genes observed in control cells was attenuated in HIF1α-depleted cells (Fig. 6G). Notably, not all BETi‑responsive metabolic genes were equally dependent on HIF1α, indicating that BET inhibition induces a partially HIF1α‑dependent glycolytic transcriptional program, likely reflecting hierarchical or parallel regulatory mechanisms downstream of BET proteins. HIF1α expression was also reduced under basal conditions and remained significantly suppressed upon BETi treatment, indicating that BETi does not rescue HIF1α expression once HIF1α is depleted (Supplementary Fig. 2H). Notably, these transcriptional changes do not reflect in a significant metabolic output at the analyzed time point (Supplementary Fig. 2I), although a modest downward trend is observed. This suggests that the glycolytic phenotype (as measured by the Lac/Ala ratio) may lag behind transcriptional regulation and could emerge more clearly at later time points. To further support that HIF1α is not a universal regulator of basal glycolysis in TNBC, but rather mediates an adaptive, BETi-induced glycolytic response in specific subtypes of TNBC cells, we evaluated the expression of Fructose-Bisphosphatase 1 (FBP1), a negative regulator of glycolysis [30, 31] and reported to interact with HIF1α and impair HIF-mediated transcriptional programs associated with glycolytic reprogramming [32]. FBP1 expression was reduced upon BETi in MB231 and BT549 cells, while remaining largely unchanged in Hs578t (Fig. 6H, left). HIF1α silencing increases FBP1 levels in MB231 and BT549 cells and prevents its repression upon BET inhibitor treatment (Fig. 6H, right), further supporting that HIF1α is not a universal regulator of basal glycolysis in TNBC, but rather mediates an adaptive, BETi-induced glycolytic response in specific subtypes (MB231 and BT549). This is corroborated by the similar basal expression of glycolytic genes (GLUT1, HK2, ENO1, PKM2, and LDHa) and glycolytic regulators (FOSL1 [33] and FBP1) (Supplementary Fig. 3A). Collectively, these findings establish that BETi induces a HIF1α‑dependent, cMYC‑independent glycolytic reprogramming in cMYC‑high TNBC cells, a response absent in Hs578t cells that highlights the metabolic heterogeneity of TNBC and reveals a potentially targetable, BETi‑induced pseudohypoxic vulnerability in susceptible tumors. We summarize all the results obtained in Fig. 6I.

## Discussion

Triple-negative breast cancer remains one of the most clinically challenging breast cancer subtypes due to its aggressive behavior, lack of targeted therapeutic options, and pronounced transcriptional and metabolic heterogeneity and plasticity. Although BET inhibitors have emerged as promising agents targeting oncogenic transcription programs, including cMYC-driving networks, their clinical efficacy as single agents has been limited by adaptive resistance mechanisms and dose-limiting toxicities. In this study, we identify a previously unappreciated metabolic adaptation to BET inhibition in a defined subset of TNBC and demonstrate how this adaptive response creates a targetable vulnerability that can be exploited through rational combination therapy. Our results demonstrated that basal BRD4 and cMYC expression do not strictly predict overall sensitivity to BETi, but rather shape the qualitative nature of the response. While Hs578t cells show a predominantly cytostatic phenotype, MB231 and BT549 cells undergo pronounced apoptotic responses accompanied by mitochondrial dysfunction and a compensatory metabolic response that ultimately dictates therapeutic outcome. The central discovery of this work is that BET inhibition induces a delayed, adaptive metabolic rewiring toward glycolysis in cMYC-high TNBC cells. In contrast, Hs578t cells, which display a strong basal glycolytic bias, fail to engage this compensatory response. We further establish that the intrinsic basal metabolic phenotype determines sensitivity to the glycolysis inhibitor 2-DG. Hs578t cells were the most sensitive to 2-DG, while MB231 and BT549 showed reduced susceptibility. Mechanistically, we identify that HIF1α stabilization is required for the BETi–induced glycolytic transcriptional reprogramming in cMYC-high cells. BETi treatment promotes HIF1α accumulation and activity in MB231 and BT549 cells through post-transcriptional mechanisms, recapitulating a pseudohypoxic state that supports Warburg-like metabolism independently of cMYC. HIF1α depletion attenuates the induction of glycolytic genes, including GLUT1, PKM2, and LDHA, confirming its functional relevance. However, this transcriptional effect is only partially reflected at the metabolic level at early time points, where Lac/Ala ratios show modest or non-significant changes. Although the Lac/Ala ratio provided a useful longitudinal surrogate of glycolytic activity, we acknowledge that it does not directly quantify glycolytic flux; however, it suggests that this adaptive response is not immediate at the metabolic level, as transcriptional rewiring precedes measurable changes in metabolic output, indicating a temporal uncoupling between gene expression and metabolite flux. This is consistent with the inability of Hs578t cells to stabilize HIF1α upon BETi, reinforcing the concept that HIF1α-dependent plasticity underlies inducible, rather than basal, glycolytic control. Future studies integrating real-time extracellular flux measurements will further refine the metabolic characterization of BETi-induced adaptations in TNBC models. This finding also highlights an unanticipated consequence of epigenetic therapy: targeting transcriptional inhibition can actively reprogram cellular metabolism rather than simply suppress it. Consequently, these cells develop a new, targetable vulnerability: they become exquisitely sensitive to the combination of BETi and the glycolysis inhibitor 2-DG. This combination yields an additive effect, significantly impairing mitochondrial metabolic activity and driving apoptotic cell death beyond what either agent can achieve alone. These observations caution against uniform therapeutic strategies and emphasize the importance of metabolic context in determining treatment response. Critically, the HIF1α-driven metabolic rewiring induced by BETi in cMYC-high TNBC cells creates a novel, targetable vulnerability, rendering these cells exquisitely sensitive to the combination of BETi and the glycolysis inhibitor 2-DG. While neither BETi nor 2-DG alone fully suppressed cell viability, their combination produced a robust additive effect, significantly impairing mitochondrial metabolic activity and promoting apoptotic cell death beyond what either agent could achieve alone. Notably, this cooperative interaction was most evident in apoptosis assays rather than proliferation readouts, indicating that the primary benefit of the combination lies in overcoming survival mechanisms rather than enhancing cytostatic effects. Importantly, this strategy was ineffective in Hs578t cells, which appeared to engage alternative metabolic pathways, further supporting a HIF1α-dependent adaptive mechanism, again underscoring the metabolic plasticity and heterogeneity of TNBC. This finding strengthens the model of a coordinated transcriptional program driving glycolytic adaptation. These interpretations align with the growing understanding of TNBC as a disease defined by diverse metabolic vulnerabilities beyond glucose metabolism. For instance, recent studies have highlighted a critical interplay between exogenous cystine availability and glutamine dependence, showing that cystine uptake can modulate glutamine addiction and, under glutamine-limited conditions, even contribute to metabolic stress rather than supporting cell survival [34]. Furthermore, lipidomic profiling has identified distinct signatures, including increased levels of phosphatidylethanolamine, that differentiate aggressive TNBC models such as MB231 from luminal breast cancer cells like MCF7, supporting the idea that lipid remodeling is a key feature of TNBC metabolic identity [35]. These interpretations align with the growing understanding of TNBC as a disease characterized by diverse and adaptable metabolic vulnerabilities extending beyond glucose metabolism [36, 37], and further highlight that such metabolic dependencies are not solely intrinsic but can also be dynamically induced by epigenetic therapies such as BET inhibition. The therapeutic implications of this work are substantial, as it provides a dual-purpose biomarker strategy and highlights programmed cell death as the key therapeutic outcome. We propose that high BRD4/cMYC expression may identify tumors predisposed not only to BETi sensitivity but also to HIF1α-mediated adaptive rewiring. Rather than serving as a static predictor of response, BRD4/cMYC status may define a dynamic transcriptional–metabolic state associated with inducible glycolytic dependency. This adaptive state, in turn, creates a vulnerability that can be exploited through glycolysis inhibition. The analysis of TCGA data provides supportive, hypothesis-generating evidence for the clinical relevance of this concept. Within the limitations of this heterogeneous cohort, it suggests that a BRD4-high transcriptomic signature is associated with a glycolytic gene program (ENO1, GLUT1). This supports a model in which tumors exhibiting a BRD4-high/glycolytic signature may be more likely to engage the HIF1α-driven adaptive response we identified. However, given the mixed subtype composition and treatment variability of TCGA-BRCA, these observations do not allow patient stratification or prediction of therapeutic response. In this context, BRD4 could represent a candidate biomarker of a transcriptional–metabolic state, rather than a definitive predictor of response, that may be associated with an adaptive vulnerability to BET inhibition. This vulnerability could potentially be exploited through combination strategies targeting glycolysis, such as BETi combined with 2-DG. Consistent with this hypothesis, our in vitro data show that the BETi/2-DG combination exerts additive effects in BRD4/cMYC-high TNBC models, where BETi-induced metabolic rewiring generates a targetable dependency on glycolysis. Whether this relationship translates to patients will require validation in molecularly defined TNBC cohorts. This framework moves beyond using BRD4 as a static marker of initial sensitivity, suggesting instead that it may reflect a dynamic transcriptional–metabolic state associated with adaptive vulnerability. Despite the potential relevance of these findings, several critical questions remain and pave the way for future investigation. First, elucidating the precise molecular link between BET inhibition and HIF1α protein accumulation/stabilization and the broader implications of pseudohypoxia. It would be interesting to investigate whether the BETi-induced pseudohypoxic state, while creating a metabolic vulnerability, could also inadvertently promote a more aggressive or treatment-resistant phenotype in a small, persistent subpopulation of cells. Long-term studies examining markers of cancer stem cells (e.g., CD44+/CD24-) and invasion capacity in cells that survive the combination treatment are necessary to rule out this potential risk [38]. Then, the promising in vitro data must be rigorously tested in preclinical in vivo models. Patient-derived xenografts (PDXs) stratified by BRD4/cMYC status and glycolytic signature would be the ideal platform to confirm the efficacy of the BETi/2-DG combination and to establish a therapeutic window. A critical aspect of this validation will be monitoring for potential on-target toxicities of 2-DG, particularly in glucose-sensitive tissues like the brain [39]. In conclusion, this study reveals that BET inhibition in cMYC-high cells does not simply suppress oncogenic transcription but actively reshapes cellular metabolism through HIF1α-mediated adaptive glycolytic reprogramming. This response, which involves both induction of glycolytic genes and deregulation of BETi targets, creates a targetable vulnerability to glycolysis inhibition, providing a mechanistic rationale for the pro-apoptotic effect of BETi and 2-DG combination in selected TNBC subtypes. This adaptive rewiring creates a targetable vulnerability to glycolysis inhibition, providing a mechanistic rationale for combining BET inhibitors with metabolic therapies.

## Materials and Methods

### Cell lines and treatments

The human TNBC cell lines MDA-MB-231 (MB231), Hs578t, and BT549 were obtained from ATCC, while the luminal breast cancer cell line MCF7 was purchased from Sigma-Aldrich. Cell line authentication was performed via SNP profiling (Multiplexion GmbH, Germany) in March 2026. MB231 and BT549 were cultured in DMEM high glucose with GlutaMAX (Gibco) supplemented with 10% fetal bovine serum (Gibco). Hs578t cells were maintained in DMEM high glucose with GlutaMAX containing 10% FBS and 0.01 mg/mL human insulin (Merck Sigma-Aldrich). MCF7 cells were cultured in MEM with GlutaMAX and Earle’s salts (Gibco), supplemented with 10% FBS and 1% non-essential amino acids (NEAA, Gibco). All culture media included 1% penicillin-streptomycin (Lonza). Cells were maintained at 37 °C in a humidified incubator with 5% CO₂ and routinely tested bimonthly for Mycoplasma contamination through the MycoAlert mycoplasma detection kit (Lonza). For drug assays and proliferation assays, cells were treated for 72 hours with the indicated concentrations of BET inhibitors (JQ1 or OTX-015, Merck Sigma-Aldrich and Medchem, respectively; 0.2–5 μM) or 2-deoxy-D-glucose (2-DG; Merck Sigma-Aldrich; 0.2–5 mM). For combination treatments, cells were co-treated with optimized doses of BETi (0.2–0.5 μM) and 2-DG (2 mM for MB231, 1.5 mM for BT549, 0.5 mM for Hs578t, and 1 mM for MCF7) for 72 hours. For mitochondrial metabolic activity assessment, cells were exposed to treatments for 72 hours, with the exception of time-course experiments for IC50 determination, which were performed at 24, 48, and 72 hours. Apoptosis assays were conducted after 72 hours of treatment. For gene expression analysis by RT-qPCR, cells were treated with 1 μM BETi for 6, 24, or 48 hours, as indicated in the respective figures. Protein expression analysis was performed after 24 hours of BETi treatment. For hypoxia-mimetic experiments, cells were treated with 1 μM BETi and/or 100 μM CoCl₂ for 24 hours prior to analyses. For actinomycin D experiments, cells were pre-treated with BETi for 24 hours, followed by 1 μg/mL actinomycin D for the indicated time points. Summarized methods, exact sample size (*n*) for each experimental group/condition, biological replicates, and statistical method are listed in Table 1.

**Table 1:** RT-qPCR primers for gene expression analyses and summary of the experiments, with the relative treatment, duration, sample size, and applied statistical methods.

Target	*Forward*	*Reverse*
ACTB	*TGCGTTACACCCTTTCTTGA*	*AAAGCCATGCCAATCTCATC*
SLC2A1/GLUT1	*TTCACTGTCGTGTCGCTGTT*	*TGAGTATGGCACAACCCGC*
GLUT3	*GGACGTGGAGAAAACTTGCTG*	*TCAGAGCTGGGGTGACCTTC*
GLUT4	*AGCACCGCAGAGAACACAG*	*GTCGGGCTTCCAACAGATAG*
HK1	*TCCAACAGCCACAGTCAAGA*	*GGGCAATGAAATCTCCCTTT*
HK2	*TCTATGCCATCCCTGAGGAC*	*AAACCCAGTGGGAGCTTCTT*
ALDOA	*GGAAAAGGGCAGGGGTCATTA*	*AGTAGCAAGTTCCTGCGGC*
GAPDH	*TCAAGAAGGTGGTGAAGCAGG*	*ACCAGGAAATGAGCTTGACAAA*
ENO1	*TGATCGAGATGGATGGAACA*	*CGCCATTGATGACATTGAAC*
PKM2	*TCGCATGCAGCACCTGATT*	*CCTCGAATAGCTGCAAGTGGTA*
LDH-A	*AGCCCGATTCCGTTACCT*	*CACCAGCAACATTCATTCCA*
SLC16A1/MCT1	*GGCTTGATTGCAGCTTCTTT*	*CAATCATGGTCAGAGCTGGA*
SLC16A4/MCT4	*AAGAGCCATTGTATGTCTGGG*	*GGGACTTGCCAGTTTCTTTG*
CPT1A	*TGCTTTACAGGCGCAAACTG*	*GCTGGATGGTGTCTGTCTCC*
ACADVL	*CAGGAGCCGTGAAGGAGAAG*	*GGCACCCGTACTCCATCAAA*
ECHS1	*GCCTCGGGTGCTAACTTTGA*	*GCCATCGCAAAGTGCATTGA*
COX5B	*GTCCCCTCCATCTCCAACAA*	*TTGTGCAGCCAAAACCAGAC*
GLS	*TTTTGTTTGCTGCATATACTGGA*	*ATAGTCCCGCTGTTCCATGT*
GLUD1	*AGGAATGACACCAGGGTTTG*	*CCAACAGCAATACATTTAGCACC*
HIF1α	*ACACACAGAAATGGCCTTGTG*	*TCTTCCTCGGCTAGTTAGGGT*
NDRG1	*CGCCAGCACATTGTGAATGAC*	*TTTGAGTTGCACTCCACCACG*
cMYC	*ATTCTCTGCTCTCCTCGACG*	*AGCCTGCCTCTTTTCCACA*
FOSL1	*GTACGTCGAAGGCCTTGTGA*	*GTTCCTTCCTCCGGTTCCTG*
FBP1	*ACATCGATTGCCTTGTGTCC*	*CCACCAAAATGAACTCCCCG*

Assay	Figure	Treatment	Duration	Doses	Endpoint time	Sample size (*n*)	Sample collection	Statistical methods
Proliferation (BETi)	Fig. 1	BETi	72 h	0.2uM->5uM	72 h	*n* = 5	biological replicates	ttest, *P* value, error bars as s.e.m.
Mitochondrial activity (BETi)		BETi	72 h	0.2uM->5uM	72 h	*n* = 7		
Apoptosis (BETi)		BETi	72 h	0.2uM->5uM	72 h	*n* = 4		
Protein expression (WB)		wt cells, no treatments				*n* = 3		not performed
Metabolic analysis	Fig. 2	wt cells, no treatments				*n* = 3		not performed
Gene expression (RT-qPCR)		BETi	48 h	1uM	6, 24, 48 h	*n* = 4		ttest, *P* value, error bars as s.e.m.
Protein expression (WB)		BETi	24 h	1uM	24 h	*n* = 3		
IC50 time-course	Fig. 3	2-DG	72 h	several doses	24, 48, 72 h	*n* = 3		
Proliferation (2-DG)		2-DG	72 h	0.2mM->5 mM	72 h	*n* = 3		
Mitochondrial activity (2-DG)		2-DG	72 h	0.2mM->1 mM	72 h	*n* = 10		
Proliferation (combination)	Fig. 4	BETi + 2-DG	72 h	Cell line-dependent	72 h	*n* = 2 MB231, BT549; n = 4 Hs578t		
Mitochondrial activity (combination)		BETi + 2-DG	72 h	Cell line-dependent	72 h	*n* = 6		
Apoptosis (combination)		BETi + 2-DG	72 h	Cell line-dependent	72 h	*n* = 4		
Gene expression (RT-qPCR)		BETi	48 h	1uM	48 h	*n* = 4		
Protein expression (WB)	Fig. 6	BETi ± CoCl2	24 h	1 uM ± 100 mM	24 h	*n* = 3		not performed
Gene expression (RT-qPCR)		BETi	24 h	1uM	24 h	*n* = 7		ttest, P value, error bars as s.e.m.
Gene expression (RT-qPCR)		BETi ± ActD	24 h	1 uM ± 1 μg/mL	24 h + 6 h	*n* = 3		
Protein expression (WB)		siHIF	24 h	none	24 h	*n* = 1		not performed
Gene expression (RT-qPCR)		BETi ± siHIF	24 h	1uM	24 h	*n* = 4		ttest, *P* value, error bars as s.e.m.

Assay	Supp figure	Treatment	Duration	Doses	Endpoint time	Sample Size (*n*)	Sample collection	Statistical methods
Proliferation (BETi)	Supplementary Fig 1	BETi	72 h	0.2uM->5uM	72 h	*n* = 5	biological replicates	ttest, *P* value, error bars as s.e.m.
Mitochondrial activity (BETi)		BETi	72 h	0.2uM->5uM	72 h	*n* = 7		
Protein expression (WB)		BETi	72 h	0.5uM, 1uM	72 h	*n* = 2		not performed
Metabolic analysis		wt cells, no treatments				*n* = 3		ttest, *P* value, error bars as s.e.m.
Gene expression (RT-qPCR)	Supplementary Fig 2	BETi	24 h	1uM	24 h	*n* = 4		
Mitochondrial activity (2-DG)(MCF7)		2-DG	72 h	0.2mM->0,5 mM	72 h	*n* = 10		
Proliferation (combination)		BETi + 2-DG	72 h	Cell line-dependent	72 h	*n* = 3		
Protein expression (WB)		BETi ± CoCl2	24 h	1 uM ± 100 mM	24 h	*n* = 2		not performed
Protein expression (WB)(HIF1a)		BETi	24 h	1uM	24 h	*n* = 3		not performed
Gene expression (RT-qPCR)		BETi ± ActD	24 h	1 uM ± 1 μg/mL	24 h + 6 h	*n* = 3		ttest, *P* value, error bars as s.e.m.
Gene expression (RT-qPCR)		BETi ± siHIF	24 h	1uM	24 h	n = 4		
Metabolic lac/ala ratio		BETi ± siHIF	24 h	1uM	24 h	*n* = 4		
Gene expression (RT-qPCR)	Supplementary Fig 3	wt cells, no treatments				*n* = 4		not performed

### siRNA transfection

For siRNA transfection: MB231 and BT549 cells were reverse-transfected with RNAiMax Lipofectamine (Thermo Scientific, Waltham, MA, USA) according to the manufacturer’s instructions. After 6 hours of transfection, 1 μM BETi was added to the cells. siRNA validation was collected 24 hours after treatment. siRNAs used were Silencer Select siRNA specific for HIF1α (ID: s6539, Thermo Scientific, Waltham, MA, USA) and Silencer Select RNAi Negative Control (Thermo Scientific, Waltham, MA, USA) at a final concentration of 20 nM.

### NMR reagents

NMR spectroscopy analysis was performed using deuterated reagents: methanol (CH_3_OH), chloroform (CHCl_3_), and D_2_O were purchased from Cambridge Isotope Laboratories, Inc.; while 3-(trimethylsilyl)propionic-2,2,3,3-d4 acid sodium salt (TSP) was obtained from Merck & Co, Montreal, Canada.

### Cell proliferation

TNBC (MB231, Hs578t, and BT549) and luminal (MCF7) cells were seeded at 3×10³ cells/well in 96-well plates (Corning #3599). After 24 hours, cultures were treated with control (DMSO or H_2_O), BETi, or 2-DG in complete medium. Plates were transferred to an Incucyte S3 system (Essen BioScience) maintained at 37 °C/5% CO₂. Phase-contrast images (10X) or NucLight Rapid Red (Sartorius #4717) fluorescence images were acquired every 24 hours for 72 hours. Cell proliferation was quantified as a percentage of viable (fluorescence) cells (Incucyte Base Software).

### Mitochondrial metabolic activity by MTT assay

Mitochondrial metabolic activity was assessed using the MTT assay. Briefly, 3 × 10³ cells/well were plated in 96-well plates and treated as indicated. Afterward, the cells were incubated with 40 μL of MTT reagent (5 mg/mL, Merck Sigma-Aldrich) for 3 hours at 37 °C. Formazan crystals were dissolved in DMSO (100 μL/well), and absorbance was read at 560 nm using a GloMax Discover system (Promega). Data were normalized to the untreated control.

### Cell death analysis

Cell death was assessed using the Annexin V-PE Apoptosis Detection Kit (BD Biosciences, #559763) according to the manufacturer’s protocol. In brief, 1.5 × 10⁵ cells per well were seeded in 12-well plates. Both floating and adherent cells were collected, washed with PBS, and resuspended in 1X Annexin V binding buffer. Cells were stained with Annexin V-PE and 7-AAD for 15 minutes at 4 °C in the dark. Cells were washed, resuspended in 1X Annexin V binding buffer, and we proceeded with apoptosis rate measurement. Flow cytometric analysis was carried out on a FACSCanto II cytometer (BD Biosciences), gating on single cells based on FSC/SSC to exclude debris and doublets. Data were analyzed using FACSDiva software.

### Metabolomics analysis by NMR spectroscopy

Aqueous and organic metabolites were extracted from breast cancer cells treated with BETi according to the protocol previously described in [23]. The polar phase, containing water-soluble cellular metabolites, was evaporated using a rotary evaporator and subsequently lyophilized. The organic (lipid) fraction was evaporated under a stream of nitrogen gas. Both fractions of the cellular extracts were stored at –20 °C until analysis. The aqueous phase of cellular extracts and extracellular medium samples (300 μL) was reconstituted in 700 μL of deuterium oxide (D_2_O) containing 0.1 mM 3-(trimethylsilyl)propionic-2,2,3,3-d₄ acid (TSP), which served as the internal standard for NMR spectroscopy. High-resolution 1H-NMR analyses were carried out at 25 °C using a 14.1 T Bruker AVANCE Neo spectrometer (Bruker). Spectra of aqueous extracts were acquired using the noesypr1d pulse sequence for water suppression. Data acquisition, processing, and peak area deconvolution were performed as previously described [23]. The absolute quantification of aqueous metabolites, determined by comparing the integral of each metabolite to the integral of reference standard TSP and corrected by respective proton numbers for metabolite and TSP, was expressed as nmoles/10^6^ cells and then converted into metabolite percentage (relative to total metabolites evaluated in each sample). Relative metabolite quantification was presented as the percentage of an individual metabolite among all metabolites (% Met/∑ all Met).

Multivariate principal component analyses (PCA) and Partial least squares-discriminant analysis (PLS-DA) were performed using the MetaboAnalyst 6.0 tool (https://www.metaboanalyst.ca/) to construct a relationship between groups and metabolic profiles. In this study, all PLS-DA models performed showed an R^2^ ≥ 0.8 and a Q^2^ ≥ 0.50, which are the goodness of fit and prediction of the models. Furthermore, we considered variable importance in projection (VIP) values greater than 1 to indicate important variables.

### RNA isolation and reverse‑transcription quantitative PCR assays

Total RNA was isolated using the Maxwell RSC simplyRNA Cells kit (Promega) according to the manufacturer’s protocol. cDNA synthesis was performed using up to 1 µg of RNA with Primescript RT Master Mix (Takara). RT-qPCR was carried out using GoTaq Supermix (Promega) and gene-specific primers (250 nM final concentration) on a Bio-Rad CFX96 Real-Time PCR System. Gene expression levels were analyzed using the 2^^−ΔΔCt^ method, with β-actin serving as the reference gene. Primer sequences are listed in Table 1.

### Western Blot Analysis

Cells were lysed in PLB buffer (Promega #E1941) containing protease inhibitor (MedchemExpress #HY-K0010) and phosphatase inhibitors (Thermo Scientific #78420). Protein concentrations were determined by Bradford assay (Bio-Rad), and equal amounts of protein (10-30 μg) were separated by 10% SDS-PAGE (Bio-Rad Mini-PROTEAN Tetra system) and transferred to nitrocellulose membranes (Bio-Rad). After blocking with 5% non-fat milk (Cell Signaling Technologies), membranes were incubated with primary antibodies overnight at 4 °C, followed by HRP-conjugated secondaries (GE Healthcare anti-Mouse #NXA931V and anti-Rabbit#NA934, 1:5000). Signals were developed using WESTAR ηC ultra 2.0 (Cyanagen) and captured with a ChemiDoc MP system (Bio-Rad). The primary antibodies used are: β-actin served as a loading control (#A1978, Sigma-Aldrich), BRD4 (#A301-985A100, Bethyl), PKM2 (#A303-660A, Bethyl), cMYC (#9E10 (SC40), SantaCruz BT), and HIF1α (#610959, BD Biosciences). All western blots were performed 2–4 times. Representative images were used in the figures.

### TCGA-BRCA data analysis

Clinical data and normalized gene expression profiles were obtained from the TCGA Breast Invasive Carcinoma dataset using the R TCGAbiolinks package. In survival analysis, performed by the R Survminer package, patients were grouped based on median gene expression; overall survival probability was represented by Kaplan-Meier curves, and significance was evaluated by the log-rank test. Correlations between genes were evaluated by R Corrplot package using the Spearman test.

### Statistical analysis

Comparisons between two data groups were analyzed using an unpaired two-tailed Student t-test (GraphPad Prism 9 or Excel). *P*-values < 0.05 were considered significant and indicated in the graphs by * or ^#^. *P*-values < 0.01 or < 0.001 were indicated by ** or ^##^ and *** or ^###^, respectively. All data were shown as mean and SEM. Each experiment was replicated two to five times.

## Supplementary information

Supplementary Material

Original Data

## Data Availability

Data sharing not applicable to this article as no datasets were generated or analyzed during the current study.

## References

[1] Hanahan D, Weinberg RA. Hallmarks of cancer: the next generation. Cell. 2011;144:646–74. 10.1016/j.cell.2011.02.013 21376230

[2] Vander Heiden MG, Cantley LC, Thompson CB. Understanding the Warburg effect: the metabolic requirements of cell proliferation. Science. 2009;324:1029–33. 10.1126/science.1160809 PMC2849637 19460998

[3] Warburg O, Wind F, Negelein E. The metabolism of tumors in the body. J Gen Physiol. 1927;8:519–30. 10.1085/jgp.8.6.519 PMC2140820 19872213

[4] Long JP. Targeting metabolism in breast cancer: How far we can go? WJCO. 2016;7:122. 10.5306/wjco.v7.i1.122 PMC4734934 26862496

[5] Vander Heiden MG, DeBerardinis RJ. Understanding the intersections between metabolism and cancer biology. Cell. 2017;168:657–69. 10.1016/j.cell.2016.12.039 PMC5329766 28187287

[6] Pelicano H, Zhang W, Liu J, Hammoudi N, Dai J, Xu RH, et al. Mitochondrial dysfunction in some triple-negative breast cancer cell lines: role of mTOR pathway and therapeutic potential. Breast Cancer Res. 2014;16:434. 10.1186/s13058-014-0434-6 PMC4303115 25209360

[7] Choi J, Jung WH, Koo JS. Metabolism-Related proteins are differentially expressed according to the molecular subtype of invasive breast cancer defined by surrogate immunohistochemistry. Pathobiology. 2013;80:41–52. 10.1159/000339513 22832328

[8] Lanning NJ, Castle JP, Singh SJ, Leon AN, Tovar EA, Sanghera A, et al. Metabolic profiling of triple-negative breast cancer cells reveals metabolic vulnerabilities. Cancer Metab. 2017;5:6. 10.1186/s40170-017-0168-x PMC5568171 28852500

[9] Shen N, Korm S, Karantanos T, Li D, Zhang X, Ritou E, et al. DLST-dependence dictates metabolic heterogeneity in TCA-cycle usage among triple-negative breast cancer. Commun Biol. 2021;4:1289. 10.1038/s42003-021-02805-8 PMC8595664 34785772

[10] Liu S, Li Y, Yuan M, Song Q, Liu M. Correlation between the Warburg effect and progression of triple-negative breast cancer. Front Oncol. 2023;12:1060495. 10.3389/fonc.2022.1060495 PMC9913723 36776368

[11] Kaur B, Sohrabi Y, Achreja A, Lisanti MP, Martinez-Outschoorn UE. Editorial: Hallmark of cancer: Reprogramming of cellular metabolism. Front Oncol. 2023;12:1126913. 10.3389/fonc.2022.1126913 PMC9874241 36713555

[12] Sun C, Yin J, Fang Y, Chen J, Jeong KJ, Chen X, et al. BRD4 inhibition is synthetic lethal with PARP inhibitors through the induction of homologous recombination deficiency. Cancer Cell. 2018;33:401–16.e8. 10.1016/j.ccell.2018.01.019 PMC5944839 29533782

[13] Sun Y, Han J, Wang Z, Li X, Sun Y, Hu Z. Safety and efficacy of Bromodomain and extra-terminal inhibitors for the treatment of hematological malignancies and solid tumors: a systematic study of clinical trials. Front Pharm. 2021;11:621093. 10.3389/fphar.2020.621093 PMC7870522 33574760

[14] Shu S, Lin CY, He HH, Witwicki RM, Tabassum DP, Roberts JM, et al. Response and resistance to BET bromodomain inhibitors in triple-negative breast cancer. Nature. 2016;529:413–7. 10.1038/nature16508 PMC4854653 26735014

[15] Rhyasen GW, Yao Y, Zhang J, Dulak A, Castriotta L, Jacques K, et al. BRD4 amplification facilitates an oncogenic gene expression program in high-grade serous ovarian cancer and confers sensitivity to BET inhibitors. Asselin E, editor. PLoS ONE. 2018;13:e0200826. 10.1371/journal.pone.0200826 PMC6056044 30036377

[16] Andrieu GP, Denis GV. BET proteins exhibit transcriptional and functional opposition in the epithelial-to-mesenchymal transition. Mol Cancer Res. 2018;16:580–6. 10.1158/1541-7786.MCR-17-0568 PMC5882530 29437854

[17] Sahai V, Redig AJ, Collier KA, Eckerdt FD, Munshi HG. Targeting BET bromodomain proteins in solid tumors. Oncotarget. 2016;7:53997–4009. 10.18632/oncotarget.9804 PMC5288238 27283767

[18] Sahni JM, Gayle SS, Bonk KLW, Vite LC, Yori JL, Webb B, et al. Bromodomain and extraterminal protein inhibition blocks growth of triple-negative breast cancers through the suppression of Aurora Kinases. J Biol Chem. 2016;291:23756–68. 10.1074/jbc.M116.738666 PMC5095428 27650498

[19] Civenni G, Bosotti R, Timpanaro A, Vàzquez R, Merulla J, Pandit S, et al. Epigenetic control of mitochondrial fission enables self-renewal of stem-like tumor cells in human prostate cancer. Cell Metab. 2019;30:303–18.e6. 10.1016/j.cmet.2019.05.004 31130467

[20] Li L, Meng Y, Wu X, Li J, Sun Y. Bromodomain-containing protein 4 inhibitor JQ1 promotes melanoma cell apoptosis by regulating mitochondrial dynamics. Cancer Sci. 2021;112:4013–25. 10.1111/cas.15061 PMC8486215 34252226

[21] Yan G, Luna A, Wang H, Bozorgui B, Li X, Sanchez M, et al. BET inhibition induces vulnerability to MCL1 targeting through upregulation of fatty acid synthesis pathway in breast cancer. Cell Rep. 2022;40:111304. 10.1016/j.celrep.2022.111304 PMC9523722 36103824

[22] Yang G, Zhou D, Li J, Wang W, Zhong W, Fan W, et al. VDAC1 is regulated by BRD4 and contributes to JQ1 resistance in breast cancer. Oncol Lett*.* 2019;18:2340–7. 10.3892/ol.2019.10534 PMC6676538 31452730

[23] Rossi T, Iorio E, Chirico M, Pisanu ME, Amodio N, Cantafio MEG, et al. BET inhibitors (BETi) influence oxidative phosphorylation metabolism by affecting mitochondrial dynamics leading to alterations in apoptotic pathways in triple-negative breast cancer (TNBC) cells. Cell Prolif. 2024;57:e13730. 10.1111/cpr.13730 PMC11628750 39223828

[24] Rossi T, Zamponi R, Chirico M, Pisanu ME, Iorio E, Torricelli F, et al. BETi enhance ATGL expression and its lipase activity to exert their antitumoral effects in triple-negative breast cancer (TNBC) cells. J Exp Clin Cancer Res. 2023;42:7. 10.1186/s13046-022-02571-3 PMC9817244 36604676

[25] Lehmann BD, Bauer JA, Chen X, Sanders ME, Chakravarthy AB, Shyr Y, et al. Identification of human triple-negative breast cancer subtypes and preclinical models for selection of targeted therapies. J Clin Invest. 2011;121:2750–67. 10.1172/JCI45014 PMC3127435 21633166

[26] Holliday DL, Speirs V. Choosing the right cell line for breast cancer research. Breast Cancer Res. 2011;13:215. 10.1186/bcr2889 PMC3236329 21884641

[27] Witt BL, Tollefsbol TO. Molecular, cellular, and technical aspects of breast cancer cell lines as a foundational tool in cancer research. Life. 2023;13:2311. 10.3390/life13122311 PMC10744609 38137912

[28] Zhang D, Li J, Wang F, Hu J, Wang S, Sun Y. 2-Deoxy-D-glucose targeting of glucose metabolism in cancer cells as a potential therapy. Cancer Lett. 2014;355:176–83. 10.1016/j.canlet.2014.09.003 25218591

[29] Aft RL, Zhang FW, Gius D. Evaluation of 2-deoxy-D-glucose as a chemotherapeutic agent: mechanism of cell death. Br J Cancer. 2002;87:805–12. 10.1038/sj.bjc.6600547 PMC2364258 12232767

[30] Lu C, Ren C, Yang T, Sun Y, Qiao P, Wang D, et al. A noncanonical role of Fructose-1, 6-Bisphosphatase 1 is essential for inhibition of Notch1 in breast cancer. Mol Cancer Res. 2020;18:787–96. 10.1158/1541-7786.MCR-19-0842 32041737

[31] Wang M, Huang X, Zhang D, Liu Y, Liu P. The role of fructose-1,6-bisphosphatase 1 on regulating the cancer progression and drug resistance. Discov Onc. 2025;16:346. 10.1007/s12672-025-02112-2 PMC11920503 40100307

[32] Song Q, Sui J, Yang Y, Zhang H, Ya L, Yang L. Fructose-1,6-bisphosphatase 1 in cancer: Dual roles, mechanistic insights, and therapeutic potential – A comprehensive review. Int J Biol Macromol. 2025;293:139273. 10.1016/j.ijbiomac.2024.139273 39753180

[33] Zhao G, Liu Y, Yin S, Cao R, Zhao Q, Fu Y, et al. FOSL1 transcriptionally dictates the Warburg effect and enhances chemoresistance in triple-negative breast cancer. J Transl Med. 2025;23:1. 10.1186/s12967-024-06014-9 PMC11697476 39748430

[34] Ge Z, Wallace M, Turner R, Yin M, Rooney MF, Porter RK. Metabolic interplay between exogenous cystine and glutamine dependence in triple-negative breast cancer. Cell Death Discov. 2025;11:430. 10.1038/s41420-025-02714-3 PMC12501009 41053011

[35] Estrada-Pérez AR, Bakalara N, García-Vázquez JB, Rosales-Hernández MC, Fernández-Pomares C, Correa-Basurto J. LC–MS based lipidomics depict Phosphatidylethanolamine as biomarkers of TNBC MDA-MB-231 over nTNBC MCF-7 cells. IJMS. 2022;23:12074. 10.3390/ijms232012074 PMC9603493 36292927

[36] Li J, Velaga R, Takano Y, Masuda N. Future perspective of clinical usefulness of metabolic and mitochondrial heterogeneity in triple-negative breast cancers. Int J Clin Oncol. 2025;30:2399–409. 10.1007/s10147-025-02916-w 41196523

[37] Xiao Y, Ma D, Yang YS, Yang F, Ding JH, Gong Y, et al. Comprehensive metabolomics expands precision medicine for triple-negative breast cancer. Cell Res. 2022;32:477–90. 10.1038/s41422-022-00614-0 PMC9061756 35105939

[38] O’Neill S, Porter RK, McNamee N, Martinez VG, O’Driscoll L. 2-Deoxy-D-Glucose inhibits aggressive triple-negative breast cancer cells by targeting glycolysis and the cancer stem cell phenotype. Sci Rep. 2019;9:3788. 10.1038/s41598-019-39789-9 PMC6405919 30846710

[39] Laussel C, Léon S. Cellular toxicity of the metabolic inhibitor 2-deoxyglucose and associated resistance mechanisms. Biochem Pharmacol. 2020;182:114213. 10.1016/j.bcp.2020.114213 32890467

