# Supplementary material for: BET inhibition unmasks a targetable glycolytic dependency through a HIF1α stabilization and driven transcriptional program in a defined subset of triple-negative breast Cancer
Source: Cell Death Discov. 2026 Jul 8;12:395. doi: 10.1038/s41420-026-03230-8 (PMC13631257; doi:10.1038/s41420-026-03230-8)
Supplement: Supplementary file 1 [file 41420_2026_3230_MOESM1_ESM.docx]

**Supplementary Figure 1. A.** Cell proliferation assay (Incucytes) on MCF7 cells using different concentrations (CNT, 0.2 μM, 0.5 μM, 1 μM, and 5 μM) of JQ1 (**left**) or OTX (**right**) over 72 hours of treatment (T(h)). **B.** MTT assay on MCF7 cells treated with JQ1 or OTX for 72 h. The results are presented as fold change of metabolic viability normalized to the control (CNT). BETi were used at a final concentration of 0.2 μM, 0.5 μM, 1 μM, and 5 μM. **C.** Western blot images on TNBC cells probed with antibodies against cMYC and β-Actin (bACT). MB231, BT549 and Hs578t cells were treated with two doses of BETi (0.5μM and 1μM) for 24, 48 and 72 hours. **D.** PLS-DA classification for supervised model estimated performance: accuracy, R^2^ and Q^2^ are quantitative measures of the model fit and consistency of the relative number of components of PLS-DA considered; these data were obtained through MetaboAnalyst software. The red star indicates the best classifier **E.** Variable importance projection (VIP) scores obtained for the three TNBC cells and hormone receptor positive cancer cells (0= MCF7; 1= BT549; 2= MB231; 3= Hs578t (n=3). Metabolites with VIP>0.8 are more important in providing class separation in PLS-DA analyses. The colored boxes on the right indicate the relative concentrations of the corresponding metabolite in each group under study. These data were obtained through MetaboAnalyst software. **F.** Relative quantification of [lac]/[ala] ratio in extracellular medium during cell growth from 3 to 72 h after cell seeding.

**Supplementary Figure 2. A.** qRT-PCR analysis of the glycolytic enzymes in MCF7 cells after 24 h of BETi administration. **B.** The band quantification of PKM2 expressed as arbitrary units (A.U.) in BT549 and Hs578t cells of the figure 2D. **C.** MTT assay on MCF7 cells treated with JQ1 (left) or OTX (right) and 2-DG, alone or combined, for 3 days. The results are presented as a fold change of metabolic activity normalized to the control (CNT). BETi were used at a final concentration of 0.5 μM and 2-DG at the IC50 relative concentration. The significance is calculated versus the CNT-treated cells (indicated with *) or between the treatments (indicated with #). **D**. Cell proliferation assay (Incucyte) on MCF7 cells to observe the effect of BETi (JQ1 or OTX) and 2-DG over 72 h of treatment. BETi and 2-DG were used alone or in combination according to the concentration indicated. **E.** Western blot images on TNBC cells treated with 100 μM CoCl_2_ for 24 hours, probed with antibodies against HIF1α, and β-Actin (bACT). **F.** Left, Western blot images on TNBC cells treated with 1μM of BETi for 24 h, probed with antibodies against HIF1α, and Tubulin (Tub). Right, Western blot images quantification and expressed as arbitrary units (A.U.). **G.** qRT-PCR analysis of the cMYC mRNA in TNBC cells after 24 hours of BETi administration, followed by the indicated time points of actinomycin D (ActD) treatments. **H.** qRT-PCR analysis of HIF1α in MB231 and BT549 cells after 24 hours of siCNT and siHIF1α transfection and treated for 24 hours with control and BETi (JQ1 and OTX). **I.** Fold change of [lac]/[ala] ratio in extracellular medium in MB231 and BT549 cells after 24 hours of siCNT and siHIF1α transfection and treated for 24 hours with control and BETi (JQ1 and OTX).

**Supplementary Figure 3. A.** qRT-PCR analysis of the glycolytic enzymes and glycolytic regulators in TNBC cells expressed as relative expression (2^-dCt).
